# Interpretable Multi-Label Classification for Tibiofibula Fracture 2D CT Images with Selective Attention and Data Augmentation

**DOI:** 10.3390/diagnostics14232740

**Published:** 2024-12-05

**Authors:** Chan Sik Han, Sun Woo Jeong, Hyung Won Kim, Seung Myung Choi, Keon Myung Lee

**Affiliations:** 1 Department of Computer Science, Chungbuk National University, Cheongju 28644, Republic of Korea; chatterboy@chugbuk.ac.kr (C.S.H.); swjeong@chugbuk.ac.kr (S.W.J.); 2 Department of Electronics Engineering, Chungbuk National University, Cheongju 28644, Republic of Korea; hwkim@chungbuk.ac.kr; 3 Bundang Cha Hospital, Seongnam 13488, Republic of Korea

**Keywords:** fracture classification, deep learning, computed tomographic scan image, multi-label classification, data augmentation

## Abstract

Background: Tibiofibula fractures occur across all age groups, and postoperative complications are frequent. An accurate and rapid classification methodology for these fractures could significantly assist physicians. Clinically, tibiofibula fractures occur at various locations, and the fracture types are not evenly distributed. Methods: This paper presents a deep learning model for the interpretable multi-label classification of tibiofibula fractures in two-dimensional (2D) CT scan images, addressing the challenges posed by a limited sample size and an uneven distribution of fracture types. We retrospectively collected 2494 2D CT images from 168 patients with tibia or fibula fractures. The types of fractures identified in the CT scan images were classified according to the AO/OTA fracture classification. A deep learning model was developed to classify composite fractures in 2D CT images, providing visual interpretation for each identified class. The visual interpretation was given with the saliency maps constructed by the Grad-CAM++ method. The deep learning model was trained using data augmentation techniques to address class imbalance and the limited dataset size. Results: Our experiments demonstrated that the proposed model achieved a mean average precision (mAP) of 95.71%. Conclusions: The saliency map-based visual interpretation enables the verification of whether the model provides reliable decision-making for classification.

## 1. Introduction

A tibiofibula fracture refers to a break involving both the tibia and the fibula, which are the two bones of the lower leg. The tibia is the larger and stronger of the two, bearing most of the body’s weight. The fibula is thinner and plays a role in stabilizing the ankle and supporting muscles of the lower leg. Determining the type of tibiofibula fracture in emergency clinics is important because it enables immediate and appropriate treatment, such as whether to perform surgery or apply immobilization, which can prevent complications and promote effective healing. Accurate diagnosis also guides the urgency plans and type of interventions needed, ensuring the best possible patient outcomes in a critical setting. In real-life medical settings, where doctors are often under tight schedules and high pressure, accurately diagnosing these fractures in a timely manner becomes a challenge.

Recent advancements in artificial intelligence (AI), particularly in deep learning models, have significantly enhanced medical imaging capabilities [1,2,3,4,5]. These advancements have introduced new methods for identifying various fracture types in the tibia or fibula bones [6,7]. However, they have focused on identifying fracture regions such as the proximal [8], shaft [9], and distal regions [10]. In addition, they consider binary classification, which simply determines the presence of a fracture in an image [11], or multi-class classification [12,13], identifying only one fracture type per image. These methods do not fully capture the complexity of real-world situations, where it is common for multiple fracture types to be present in an image. Additionally, there have been attempts to make an interpretable classification of fractures that helps visually explain the model’s outcome by using a variant of Grad-CAM [14,15]. Those efforts do not go far enough in providing visual interpretations for multiple fractures, focusing instead on identifying the presence of a fracture [11] or categorizing it into a specific type [12].

This study aims to address these limitations by proposing a deep learning model designed to identify and visually interpret both single and composite fracture types in the tibiofibula. We have explored the method to circumvent the issue of learning spurious features during training, ensuring it captures characteristics pertinent to specific fracture types. Furthermore, by identifying fracture regions along with fracture type labels, the developed model offers effective visual interpretation thereby enriching the model’s interpretability in clinical support.

## 2. Materials and Methods

### 2.1. Data Collection

We retrospectively reviewed 2494 CT scan images for 168 patients with tibial fractures involving the diaphysis, collected between January 2016 and December 2019 at a tertiary referral hospital in Korea. The mean patient age was 49.6 ± 8.1 (range 20–60) years. The three-dimensional (3D) reconstructed DICOM images were derived from CT scans in three anatomical planes—sagittal, coronal, and transverse views—with a slice thickness of 2 mm and image dimensions of 512×512 pixels. The 3D reconstructed CT scans were manually cropped into 2D squared images, ensuring that the entire tibia and fibula were centered and occupied approximately 50% of the square. These images were then resized to 384×384 pixels and stored as JPEG files. All images were de-identified to protect patient privacy. This study was approved by the Institutional Review Board of Chungbuk National University, Korea (IRB no. CBNU-202404-HR-0105), and the requirement for informed consent was waived due to the use of de-identified patient images.

The inclusion criteria for this study were (1) patients aged between 20 and 60 years, and (2) those with a definitive diagnosis of a tibial shaft fracture based on radiographic findings. The exclusion criteria were (1) non-displaced or minimally displaced tibial shaft fractures; (2) open growth plates; (3) severe osteoporosis; and (4) pathologic fractures.

The tibial shaft fractures and concurrent injuries were classified based on the AO/OTA fracture classification [16], a standard alphanumeric coding system for describing and classifying orthopedic fractures and dislocations. For diaphyseal fractures, the fractures were categorized as simple (Type A), wedge (Type B), or multi-fragmentary (Type C). Further subclassification included the following. Type A fractures were subdivided into spiral (group 1), oblique (group 2), and transverse (group 3). Type B fractures were divided into intact wedge (group 1) and fragmentary wedge (group 2). Type C fractures were subdivided into intact segmental (group 1) and fragmentary segmental (group 2), based on their morphology. For end-segment fractures, the fractures were classified as extra-articular (Type A), partial articular (Type B), and complete articular (Type C). The subclassifications were as follows: Type A fractures were grouped into avulsion (group 1), simple (group 2), and multi-fragmentary (group 3). Type B fractures were categorized as simple (group 1), split depression (group 2), and fragmentary (group 3). Type C fractures were further classified into simple articular–simple metaphysis (group 1), simple articular–multi-fragmentary metaphysis (group 2), and multi-fragmentary articular–multi-fragmentary metaphysis (group 3). Additional detailed descriptions, such as universal modifiers and qualifications, were not included.

Each fracture in the tibia and fibula was classified separately. Typically, CT scan images are labeled with one or more injuries, making fracture classification a multi-label problem. The classification of the dataset was performed by two orthopedic traumatologists and one radiologist specializing in musculoskeletal diseases. In cases of uncertainty, the corresponding full CT images, plain radiographs, or MRI scans were reviewed. Even after these additional evaluations, the images were excluded from the dataset if a consensus could not be reached. Across the 2494 images, a total of 3650 labels were assigned. Furthermore, the fracture regions in the images were annotated with bounding boxes, which were later used for data augmentation during model development.

Table 1 presents the distribution of fractures within the dataset according to the AO/OTA classification system. As shown in the table, there are 20 fracture types and one normal category. The images with composite fractures were counted multiple times, with each count reflecting the number of labels assigned to the images.

### 2.2. Preprocessing

**Figure 1:**
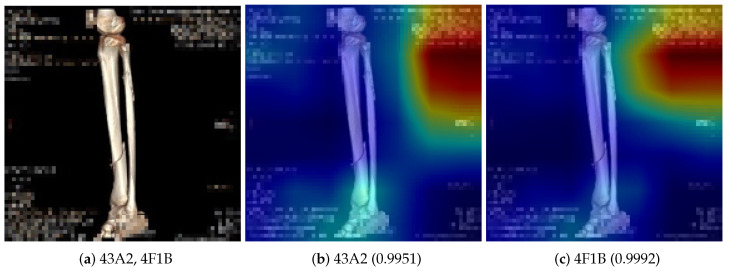
Illustration of the impact of background text during training. (**a**) An original composite fracture image with two fractures (fracture types: 43A2 and 4F1B) having blurred background text. (**b**) The saliency map for the predicted class of 43A2 type with a confidence score 0.9951. (**c**) The saliency map for the predicted class of 4F1B type of a confidence score of 0.9992. The saliency maps were generated by the Grad-CAM++ algorithm [17], which visualizes the attention areas to which the deep learning model pays attention. In the saliency maps of (**b**,**c**), the red-colored regions are the regions to which the deep learning model paid attention for classification.

Data preprocessing, along with the model architecture, is a crucial stage in deep learning model development. In the preprocessing phase, we performed background text removal and the generation of normal images. The de-identified images in the dataset contained various background text, including the date, resolution, size, compression ratio, and other metadata. The background text in images might cause the deep learning model to focus on irrelevant features. Figure 1 shows the saliency maps and the class labels produced by a deep learning model trained on the image dataset with background text. The deep learning model made the correct classification for the image in Figure 1a with background text, even though it focused on the wrong regions shown in Figure 1b,c, where no fractures were present. To prevent the model from using such spurious information, we removed the background text from all fracture images.

**Figure 2:**
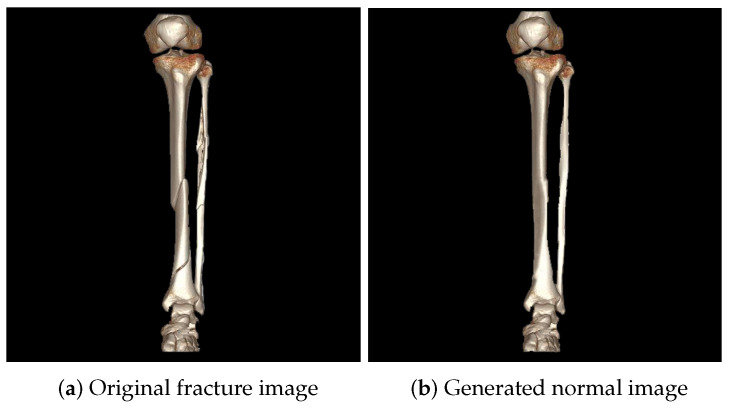
An original image and its corresponding normal image. The normal image was manually created by editing the fracture image with an image editing tool, Photoshop 2024 version 25.1.

We aimed to develop a deep learning model which focuses on fracture-specific features for fracture classification. The original dataset consisted solely of fracture images, as non-patients typically do not undergo CT imaging. Therefore, we generated normal images by editing fracture images using an image editing tool, Photoshop 2024 version 25.1. Figure 2 shows an example of a fracture image alongside its corresponding generated normal image. Our ablation study demonstrated that incorporating these generated normal images improved the model’s performance. Consequently, we used a dataset containing both fracture and generated normal images for model development.

**Figure 3:**
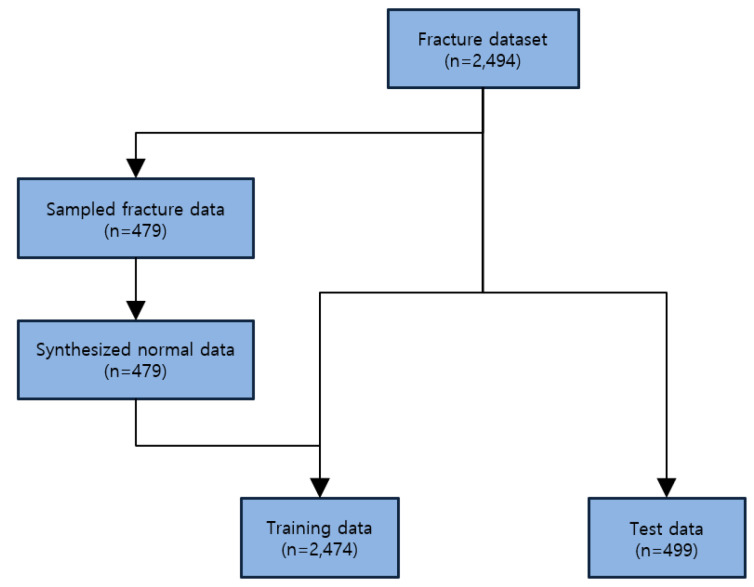
The composition of the training and test datasets.

Figure 3 illustrates the construction of the training and test datasets. The original dataset included 2494 fracture images. From this, 479 fracture images were sampled, and an equal number of normal images were synthesized, with one generated normal image for each sampled fracture image. Out of the total 2973 images (fracture and normal), 2474 were used for training, while the remaining images were reserved for testing. All synthesized normal images were included in the training set, and the test dataset comprised only fracture images.

### 2.3. Data Augmentation

**Figure 4:**
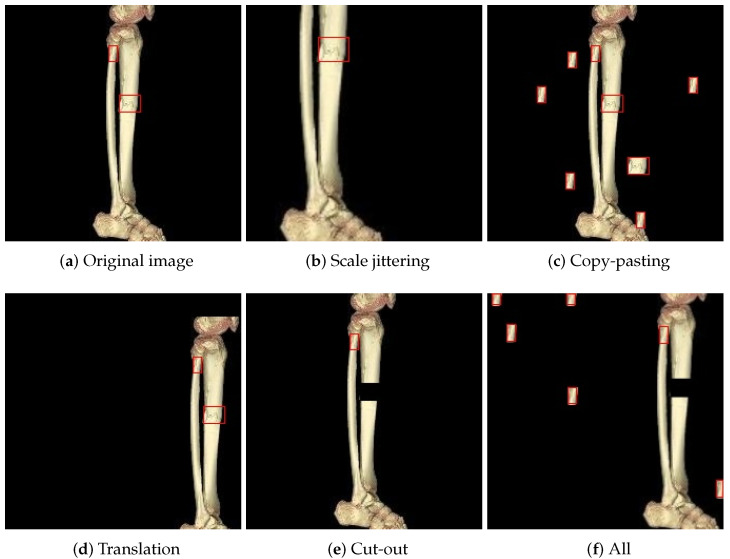
Illustration of data augmentations used in this study. The red boxes indicate the fractured areas. (**a**) An original image with red bounding rectangles for fracture regions, (**b**–**e**) the generated images when each data augmentation technique was applied to the original image, (**f**) an image obtained when all four data augmentation techniques were applied.

Data augmentation is used to artificially increase the size and diversity of a training dataset by creating modified versions of the original data [18]. These modifications help make the model more robust, improve generalization, and reduce overfitting, particularly when the training dataset is not sufficiently large. The augmentation techniques we employed included copy-pasting, scale jittering, translation, and cut-out. Figure 4 illustrates all the data augmentation techniques applied to a fracture image with two fractures in the model development as shown in Figure 4a. In the images, the red rectangles indicate the bounding boxes for the fracture regions. If all four data augmentation techniques are applied to the image in Figure 4a, the augmented image shown in Figure 4f is generated.

#### 2.3.1. Scale Jittering

The dataset contained images with varying scales, resulting in a diverse range of fracture sizes. To efficiently capture the features of fractures at different scales, we applied scale jittering [19] to the dataset. In scale jittering, an image is resized by randomly increasing or decreasing its dimensions, altering the apparent size of objects while maintaining the overall aspect ratio. After resizing, the image may be cropped to fit a specific target size or padded with black in the blank regions. As shown in Figure 1, fractures are typically small in size, so resizing an image to be too small may cause the fracture areas to disappear. Conversely, resizing an image to be too large may not be helpful for capturing fracture details. The scale jittering was applied so as to ensure that images remain neither too small nor too large. Figure 4b shows the result of scale jittering applied to the original image in Figure 4a.

#### 2.3.2. Copy-Pasting

Table 2 presents the statistics of IoU (Intersection over Union) values between images and their fracture regions for each fracture type. The IoU represents the ratio of the fracture region to the image size. According to Table 2, fracture sizes are consistently very small (i.e., around 0.01) across all fracture types. Learning the features of such small objects in images can be challenging. To enhance the likelihood of extracting meaningful features, we applied the copy-pasting technique [20].

Copy-pasting involves copying some region within an image and placing it in a random location. In our method, the regions within the bounding boxes for fractures in an image were copied and pasted at random positions in the same image. Figure 4c illustrates the result of applying copy-pasting to the original image in Figure 4a. We adjusted the number of pastes based on the size of the fracture, ensuring that smaller fractures were copied more frequently.

#### 2.3.3. Translation

**Figure 5:**
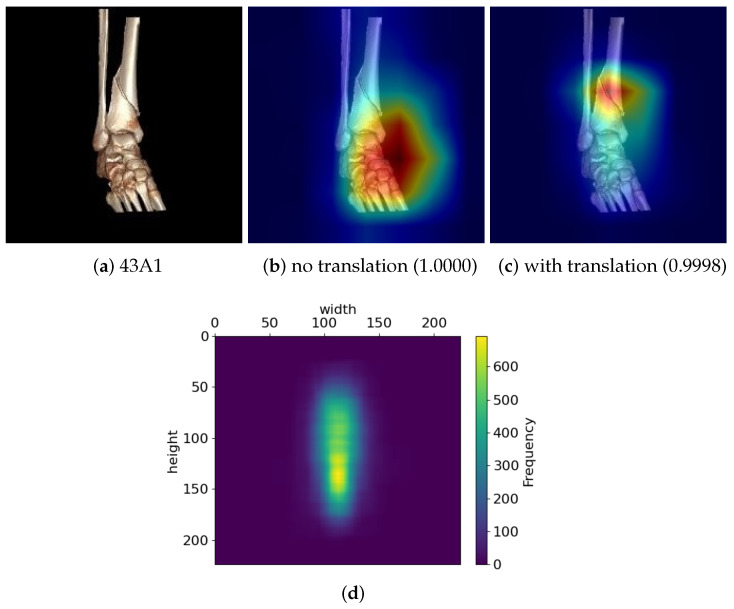
Illustration of the effect of translation for training. (**a**) A fracture image with a 43A1 type fracture, (**b**) a saliency map generated by Grad-CAM++ for the type 43A1 when a deep learning model was trained with no translation to the training data, (**c**) a saliency map for the type 43A1 when the translation was applied to the training data, and (**d**) the frequency distribution of fracture locations in the images of the training dataset.

In image data augmentation, translation involves shifting the entire image horizontally or vertically by a certain number of pixels. This technique helps models become invariant to slight shifts in object positions, improving their ability to detect features regardless of the object’s location. Figure 5 demonstrates the effects of translation on the deep learning model. Without translation, the model focused on the wrong region as shown in the saliency map generated by Grad-GAM++ of Figure 5b for the original fracture image in Figure 5a. After applying translation, the model correctly detected the fracture region as seen in Figure 5c.

Figure 5d displays the frequency distribution of fracture locations in the images of the training dataset, indicating that fractures tend to occur in similar areas across images. This tendency may lead the model to incorrectly focus on non-fracture regions when they appear in low-density areas. Applying translation helps the model more accurately detect the actual fracture regions.

#### 2.3.4. Cut-Out

Cut-out is a data augmentation technique that involves masking or cutting out random square or rectangular regions of an image with a solid color (often black) as shown in Figure 4e. This technique is used to make models more robust by encouraging them to rely on surrounding image areas for recognition and reducing overfitting [21]. Essentially, cut-out simulates real-world conditions where objects may be partially visible by introducing occlusions or missing parts in the image.

**Figure 6:**
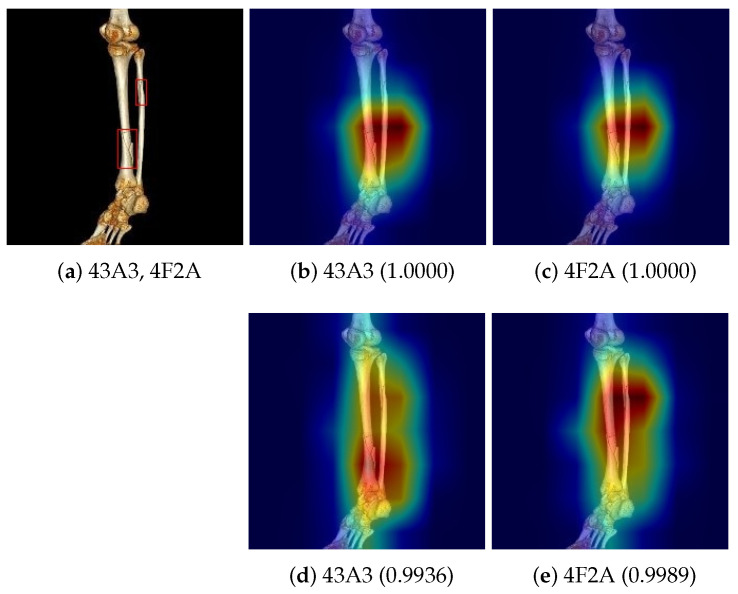
Illustration of the effect of cut-out for training. (**a**) A composite fracture image with 43A3 and 4F2A types, (**b**) the saliency map for type 43A3 when a deep learning model was trained without no cut-out data augmentation, (**c**) the saliency map for type 4F2A when a deep learning model was trained without no cut-out data augmentation, (**d**) the saliency map for type 43A3 when a deep learning model was trained with cut-out data augmentation, and (**e**) the saliency map for type 4F2A when a deep learning model was trained with cut-out data augmentation. Numbers in parentheses indicate the confidence score for the fracture type with which the trained models support the classification. The saliency maps were generated by Grad-CAM++. The saliency maps show that the cut-out data augmentation contributes to locating the fractures in the images.

For the developed deep learning model, cut-out was applied only to composite fracture images. A cut-out operation for an image with composite fractures randomly selects a bounding box for a fracture and cuts it out, while ignoring the remaining bounding boxes. Figure 6 illustrates the effect of cut-out for training. For a composite fracture image with fracture types 43A3 and AF2A as shown in Figure 6a, the deep learning model trained with no cut-out data augmentation failed to locate the correct fracture regions in the saliency map as shown in Figure 6b,d, despite making the correct classification. However, when cut-out data augmentation was applied at training, the deep learning model successfully focused more on the fracture regions as shown in Figure 6c,e.

### 2.4. Model Architecture

To classify the fracture types in the images, we used a CNN-based model with an architecture similar to ResNet-50 [22]. Table 3 outlines the architecture, which consists of 50 CNN-based feature extraction layers and a final fully connected layer with 21 nodes, corresponding to the number of fracture types in the dataset. In the table, the column *output size* indicates the dimensions of the feature map generated by each layer or block, and the column *configuration* provides the details of the convolution operations or the fully connected layer. The final feature map, sized 2048×7×7, is reduced to 2048×1×1 using a global average pooling operation.

### 2.5. Training

To train the deep learning model, we used binary cross-entropy (BCE) as the loss function, which is commonly applied in multi-label classification tasks. The BCE for class *c* is calculated as follows:

(1) $$\ell_{c}=-y_{c}\log{\hat{y}}_{c}-\left(1-y_{c}\right)\log\left(1-{\hat{y}}_{c}\right)$$

where $y_{c}$ denotes the ground-truth class label for type *c* of a given image, ${\hat{y}}_{c}=\sigma \left(z_{c}\right)$ represents the model’s output for the image, $z_{c}$ is the logit of the output node corresponding to *c*, and σ is the sigmoid function. An image with multiple fractures has multiple class labels; thus, $y_{c}$ is 1 if the image has the type label *c* and 0 otherwise. After calculating BCE for each class, their values are summed across all classes. For a batch of training images, the loss function is computed as the average of the BCE values for the images. The model was trained using the AdamW optimizer [23]. Instead of using transfer learning [24], which leverages pretrained model weights from large-scale datasets such as ImageNet [25], the model was trained from scratch with the training dataset.

### 2.6. Evaluation

To evaluate the model’s performance, we used mean average precision (mAP) as the metric. mAP measures the average precision across all classes and is widely used in multi-label classification tasks, which is calculated as follows:

(2) $$mAP=\frac{1}{C}\sum_{c=1}^{C}AP_{c}$$

where $AP_{c}$ is the average precision for class *c* and *C* is the number of classes. Average precision (AP) is a value representing the averages of all precisions, which is understood as the approximation of the area under the precision–recall curve [26].

Additionally, to visually interpret the model’s classification performance, we used Grad-CAM++ [17] for visualizing the saliency map for the regions on which the model focuses for classification. As shown in Figure 6, the Grad-CAM++ visualization helps interpret the model’s classification validity by highlighting the fracture regions that influenced the classification outcome.

### 2.7. Classification with the Model

The identification of fracture types in composite fracture images is a multi-label classification problem, where one or more class labels can be assigned to an image. Each output node in the developed model corresponds to a class label. Each node $o_{c}$ produces the value 1 when the sigmoid value ${\hat{y}}_{c}$ of the logit for its class *c*, i.e., ${\hat{y}}_{c}=\sigma \left(z_{c}\right)$, is equal to or greater than its associated threshold $\theta_{c}$, and 0 otherwise as follows:

(3) $$o_{c}=\left\{\begin{array}{c} 1,\;{\hat{y}}_{c}\geq \theta_{i}\\ 0,\;\mathrm{otherwise} \end{array}\right.$$

The threshold $\theta_{c}$ for each class *c* was determined as the value that maximized the classification accuracy for that class in the training dataset. Additionally, the saliency map generated by Grad-CAM++ was provided as a visual interpretation of the classification output.

## 3. Results

### 3.1. Setup for Model Training

The proposed method for fracture classification was implemented using PyTorch 1.13.1 and Python 3.8. The model was trained and executed on an NVIDIA RTX A6000 GPU with 48 GB of memory, running driver version 530.30.02 and CUDA version 12.1. A random seed of 20240125 was used for shuffling the dataset before splitting it into training and test sets, with an 80:20 split ratio.

In the data augmentation stage, the following setup was applied: For copy-pasting, the number of copy-pasting operations was determined based on the IoU of the fractures. If the IoU of a bounding box was less than 0.003, 5 to 10 copies were randomly made; for the IoU values less than 0.02, 0 to 3 copies were made; otherwise, 0 to 1 copy was made. Scale jittering was performed with a scale randomly selected from the range of −10% to 10%. If the scaled image became smaller than the original size, zero-padding corresponding to a black color was applied to the blanks in the bottom and right regions to match the original image size. If the image became larger, random cropping was applied to fit the original size. Translation involved shifting the image along the X or Y axis (or both) by a random number of pixels, ensuring that the fracture regions did not disappear in the translated image. Cut-out was applied only to images containing more than one fracture, with each fracture region having a 95% probability of being cut out and filled with a value of 0 (black pixels).

The batch size was set to 32, and the learning rate was 1 × 10^−3^. The AdamW optimizer [23] was used for training. To address the limited number of training samples and prevent the model from learning spurious features, diverse training samples were generated using data augmentation techniques. A large number of epochs were set during training to ensure the model captured informative features from the varied training samples. The model was trained over 1000 epochs in all experiments, without early stopping.

### 3.2. Results

We evaluated the performance of the developed model from two perspectives: first, how accurately the developed model makes classifications on fracture types, and second, how well the generated saliency maps highlight the fractures corresponding to the fracture types.

To enhance the model’s performance, we applied five data augmentation techniques: the addition of normal images, copy-pasting, scale jittering, translation, and cut-out. An ablation study was conducted to assess the contribution of each technique to the overall performance. As shown in Table 4, we trained six deep learning models with different combinations of augmentation techniques (A to F). In each combination, except for (F), one augmentation technique was excluded.

Table 4 presents the mAP scores for the trained models with different combinations of augmentation techniques. The best performance, with a mAP of 96.67, was achieved by the model corresponding to combination (E), where cut-out was not applied.

**Figure 7:**
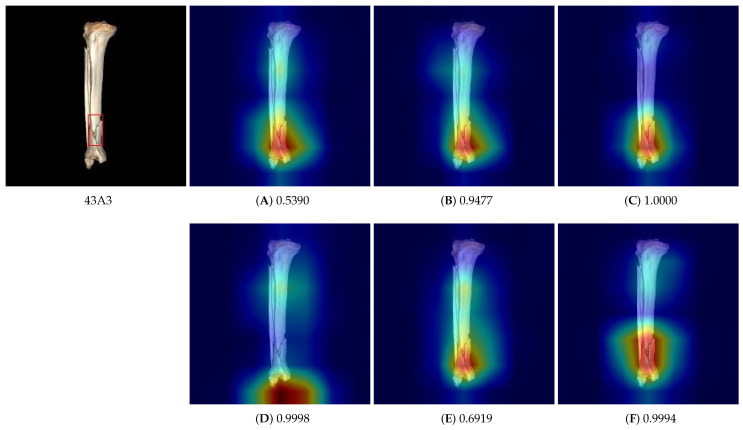
The saliency maps of the six trained models for a fracture image of type 43A3 shown in the leftmost image of the first row. In the saliency maps (**A**–**F**), the red-colored regions indicate the areas to which the trained models pay attention. The models in columns (**A**–**F**) were trained using the data augmentation techniques corresponding to the respective columns in Table 4.

In the classification of fracture types, it is important not only to achieve high accuracy (i.e., high mAP) but also to ensure that the model focuses on the fracture regions in the saliency maps. This is crucial for providing visual interpretations that medical personnel can refer to when using the classification information in practice. Hence, we examined all the saliency maps produced for the fracture images in the training dataset to determine which models were the best. Figure 7 shows the saliency maps for the six trained models for an image of type 43A3.

According to the mAP scores in Table 4, the model trained without the cut-out data augmentation (corresponding to column (E)) achieved the highest mAP. However, when we examined the saliency maps produced by the trained models, the model for column (E) did not always provide the best results. Figure 7 shows that the model corresponding to column (F) produced a better saliency map than the others. Despite the models for columns (D) and (E) having higher mAP scores, we selected the model corresponding to column (F), which used all the data augmentation techniques because it provided more consistent saliency maps. Table 5 presents the performance of the final selected model on the test dataset, where $F1_{\theta =0.5}$ indicates the F1-score evaluated at a threshold 0.5 and AP denotes the average precision. The F1-score is computed as 2(precision×recall)/(precision+recall), where precision is computed as (truepositives)/(truepositives+falsepositives) and recall as (truepositives)/(truepositives+falsenegatives). The developed model achieved an average F1-score of 0.8796 at a threshold of 0.5 and a mAP of 0.9571.

**Figure 8:**
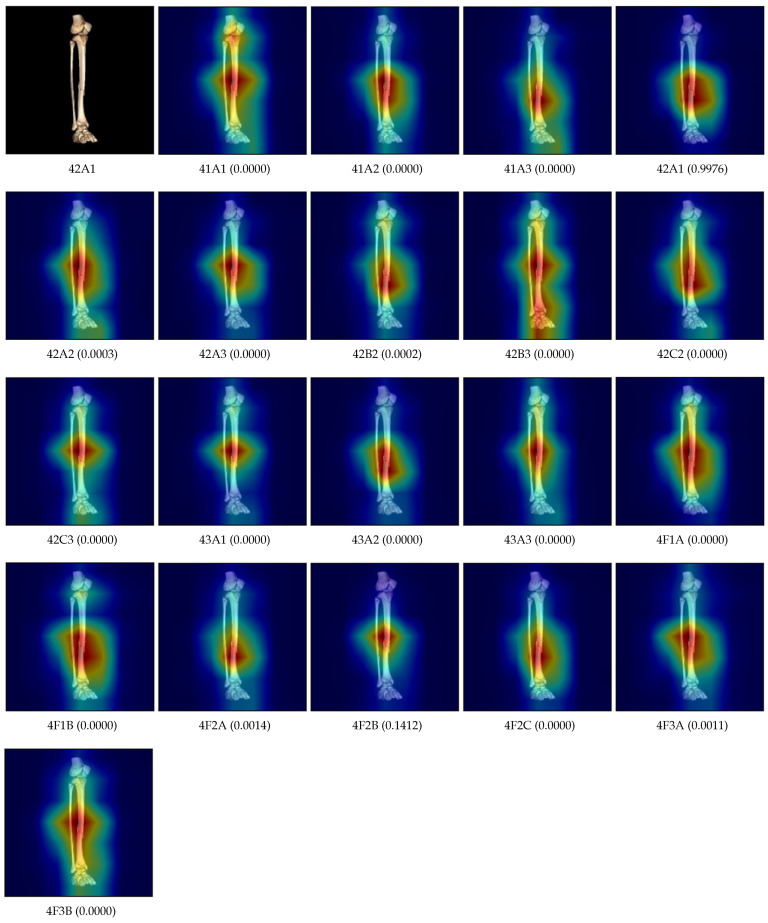
The saliency maps and confidence scores for each class label, generated by the developed model for a fracture image. Red-colored regions indicate the areas of the image to which the model pays attention for each corresponding class label. The number in parentheses represents the confidence score for that label.

Figure 8 shows the saliency maps generated by the developed classification model for each fracture type, given a single fracture image of type 42A1. The proposed model assigned a confidence score of 0.9976 for 42A1 and correctly focused on the region where the fracture was present. For all other fracture types, except for 42A2, 42B2, 4F2A, 4F2B, and 4F3A, the model assigned a confidence score of 0. The confidence scores for 42A2, 42B2, 4F2A, 4F2B, and 4F3A are significantly lower compared to 42A1. Additionally, in the saliency maps for all other fracture types, the regions corresponding to 42A1 are also highlighted.

**Figure 9:**
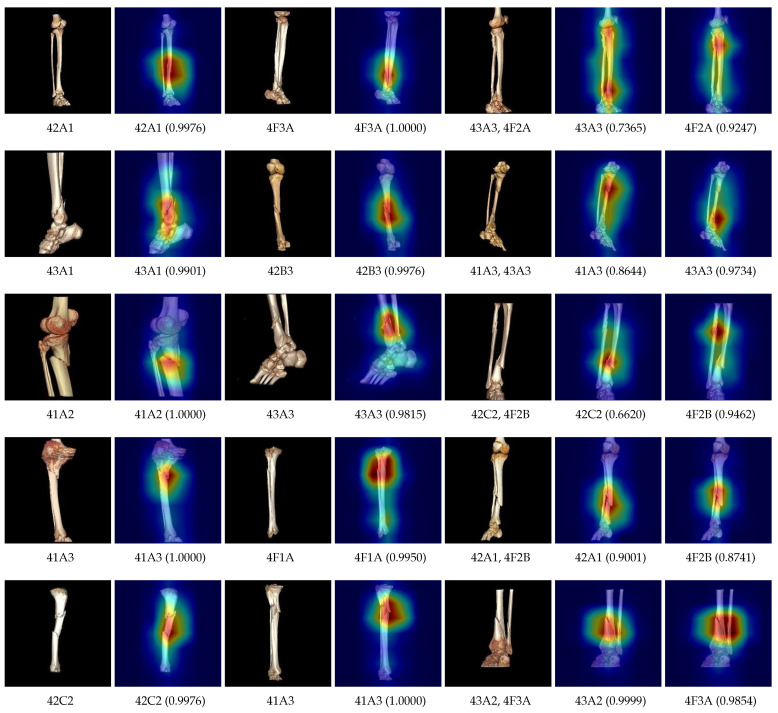
Some fracture images from the test dataset and their saliency maps and confidence scores generated by the developed model. The images with a black background are the original fracture images, labeled with their ground-truth fracture types. The colored images represent the corresponding saliency maps, displaying their predicted type label and confidence score. Images with composite fractures are assigned multiple type labels.

Figure 9 displays the saliency maps and confidence scores for some test images. The images with a black background are the original test dataset images, while their corresponding colored images highlight the saliency maps. These colored saliency maps serve as visual interpretations of the input fracture images. The labels for the saliency maps include the fracture type and its confidence score. To assist non-specialist doctors, we made the developed model produce textual explanations for class labels with confidence scores greater than or equal to their respective thresholds, in addition to the class labels and their confidence scores. For example, for the class label ‘42A1’, the model generates the following message: *a simple [A] spiral [1] fracture of the tibial shaft [42]*. Table A1 contains the full set of messages for each fracture type.

Additionally, we evaluated several advanced backbone models, including SE [27], CBAM [28], EfficientNet [29], Vision Transformer (ViT) [30], and Swin Transformer [31]. Specifically, for SE and CBAM, we implemented SE-ResNet-50 and CBAM-ResNet-50, both derived from the ResNet-50 architecture. For EfficientNet, we selected EfficientNet-B4, EfficientNet-B5, and EfficientNet-B7. For Vision Transformer, we used ViT-S/8 [32] and ViT-B/16, and for Swin Transformer, we included Swin-T and Swin-B. These models were trained on the same dataset, and their performance (measured by mAP) and interpretability were compared.

Table 6 presents the number of parameters and performance evaluation results for ResNet-50 and the other advanced models. ResNet-50 achieved the best performance, followed by EfficientNet-B4, which showed the second-best results. Attention-based modules such as the SE module and CBAM module, incorporated into SE-ResNet-50 and CBAM-ResNet-50, respectively, demonstrated lower performance compared to ResNet-50. Among the EfficientNet models, EfficientNet-B4, the smallest model in this experiment, exhibited strong performance. However, increasing the model size in the EfficientNet family led to a decline in performance. A similar trend was observed with Vision Transformer and Swin Transformer models, where relatively smaller variants achieved better performance. Specifically, increasing the model size resulted in decreased performance, akin to the pattern observed in EfficientNet. For Vision Transformer models, there was a notable performance gap between these models and others. Swin Transformer models, while not as distinctly different from other models as the Vision Transformer, exhibited relatively lower performance overall than the CNN models.

**Figure 10:**
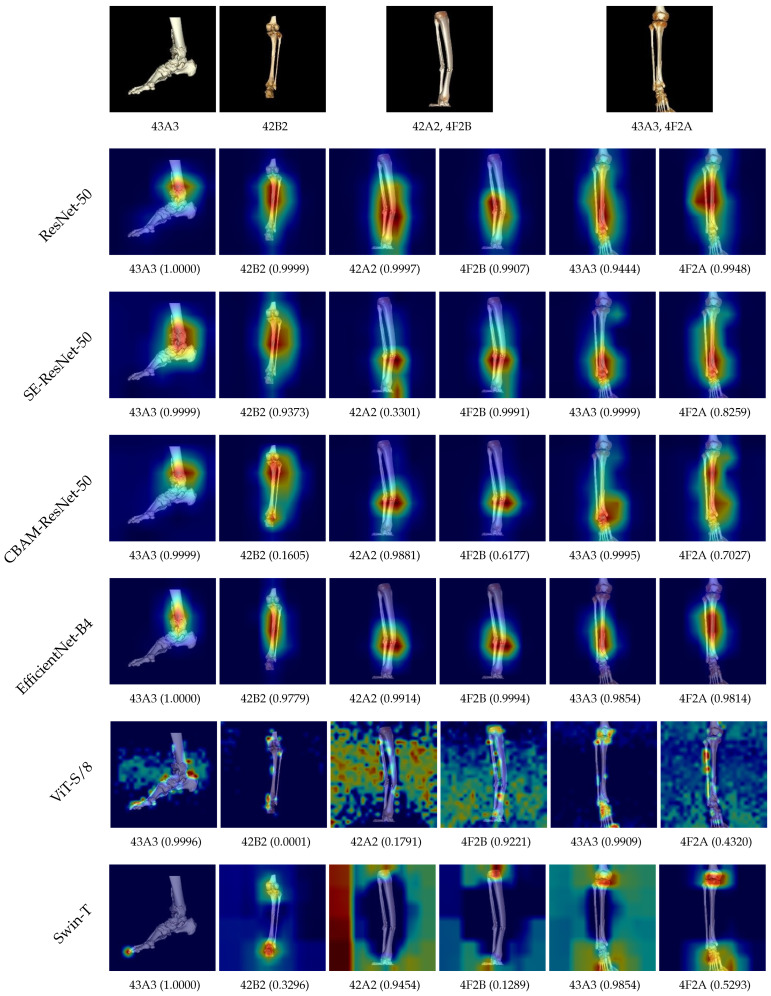
Visual explanations from ResNet-50 and other advanced models.

Figure 10 presents the visual interpretation results for ResNet-50 and other advanced models. For this analysis, we selected two single-fracture images and two composite fracture images from the test dataset. Among the models, EfficientNet-B4 was chosen to represent the EfficientNet family, ViT-S/8 was used for the Vision Transformer, and Swin-T was selected for the Swin Transformer.

ResNet-50 demonstrates a strong capability to focus on fracture-related regions across all image samples. In comparison, the other models exhibit less precise attention. While SE-ResNet-50, CBAM-ResNet-50, and EfficientNet-B4 accurately highlight fracture regions in single-fracture images, they struggle to focus on relevant areas in composite fracture images. Vision Transformer and Swin Transformer models, however, fail to adequately attend to fracture-related regions in any of the samples. Overall, CNN-based models consistently demonstrate better attention to fracture areas than Transformer-based models.

Through experiments conducted on ResNet-50 and other advanced models, we assessed the performance and interpretability of each model. ResNet-50 consistently outperformed all other models in both metrics, demonstrating its superiority. Overall, CNN-based models demonstrated better results than Transformer-based models in both performance and interpretability for the bone fracture image classification.

## 4. Discussion

### 4.1. Discussion with Previous Studies

In tibiofibula fracture classification, research has primarily focused on classifying specific regions of the tibia or fibula, such as the proximal, shaft, and distal areas. In this study, we addressed the classification of fractures across the entire tibiofibula and demonstrated that the developed model can accurately classify fractures according to the AO/OTA taxonomy. In addition, the model provides saliency maps along with confidence scores and textual comments, allowing non-specialist doctors to interpret the classification results both visually and textually.

Most fracture classification methods utilize binary classification to detect the presence of fractures, with the goal of supporting fracture identification in cases that experts may overlook during the diagnostic process. Furthermore, some studies perform multi-class classification to identify a specific fracture type from multiple candidates. Our work extends this approach by conducting multi-label classification, which can suggest multiple classes to address the complex fracture situations that occur in reality. Additionally, our method provides saliency maps for visual interpretation, helping non-expert doctors understand the classification results with the aid of textual explanations.

### 4.2. Limitations

The amount of training data was limited, and issues with class imbalance were observed, particularly affecting the evaluation of certain fracture types. This limitation challenges the model’s ability to accurately generalize across all types of tibiofibula fractures, potentially impacting its diagnostic accuracy.

Due to the scarcity of data, a comprehensive classification study encompassing all possible fracture types of the tibiofibula could not be conducted. Instead, our research focused on multi-label classification for 21 classes, including the normal class. This constraint limits the study’s scope and indicates the need for more extensive data collection to develop a model that covers all fracture types comprehensively.

The study’s reliance on single-view images introduces inherent limitations. This approach may not capture the complete anatomical context of fractures, suggesting that future research should consider utilizing multi-views or 3D imaging techniques to overcome these limitations and improve fracture classification accuracy.

### 4.3. Future Works

The 2D input images used in this study were acquired from 3D CT images in different views like AP, lateral. We utilized a single view approach, using each 2D image individually to train and evaluate the model. Some research has shown that a multi-view approach, which utilizes multiple images with different views, leads to performance improvement [33,34]. Hence, we plan to conduct research on applying the multi-view approach to our proposed method.

To support diagnosis, accurate interpretation is necessary; thus, research to improve interpretability is required. In this study, we enhanced the model’s interpretability using fracture region-guided data augmentations. Previous research has improved interpretability using attention-guided networks [15,35]. Since our proposed data augmentation can be combined with other existing research, we will conduct studies to integrate methods for improving interpretability.

## 5. Conclusions

This study presented a CNN-based deep learning model developed for the multi-label classification of tibiofibula fractures and concurrent injuries, addressing challenges posed by a limited training dataset and imbalanced class distribution. To overcome these constraints, we explored various data augmentation techniques to enhance the model’s performance for the multi-label classification of composite fractures. The developed model not only provides fracture types and their associated confidence scores for fracture images but also generates saliency maps and textual descriptions for the identified fracture types, aiding non-expert doctors in understanding the model’s classification results. However, future work is needed to develop a multi-label classification model with a larger dataset and a more balanced distribution of fracture types.

## Figures and Tables

**Table 1:** Distribution of fractures according to the AO/OTA classification system in the dataset.

Fracture Type	Training Dataset (*n* = 2474)	Test Dataset (*n* = 499)
41A1	12	3
41A2	34	14
41A3	69	15
42A1	371	93
42A2	112	35
42A3	43	13
42B2	222	43
42B3	114	32
42C2	63	24
42C3	61	12
43A1	207	52
43A2	149	34
43A3	198	57
4F1A	151	32
4F1B	155	36
4F2A	200	56
4F2B	362	85
4F2C	4	2
4F3A	243	63
4F3B	153	26
normal	479	0

**Table 2:** Statistics of the IoU between images and the fracture regions for each fracture type.

Fracture Type	Min IoU	Max IoU	Mean IoU	Variance IoU
41A1	0.0063	0.0198	0.0133	3.2052
41A2	0.0034	0.0456	0.0193	0.0001
41A3	0.0023	0.1070	0.0330	0.0007
42A1	0.0014	0.0580	0.0135	9.8580
42A2	0.0013	0.0385	0.0083	4.6640
42A3	0.0022	0.0164	0.0054	7.6071
42B2	0.0019	0.0701	0.0125	0.0001
42B3	0.0029	0.0380	0.0132	6.3476
42C2	0.0024	0.0374	0.0181	8.1315
42C3	0.0039	0.1022	0.0296	0.0003
43A1	0.0026	0.0933	0.0227	0.0002
43A2	0.0031	0.1161	0.0283	0.0008
43A3	0.0033	0.1116	0.0225	0.0004
4F1A	0.0004	0.0206	0.0044	1.1776
4F1B	0.0005	0.0740	0.0103	0.0002
4F2A	0.0002	0.0602	0.0068	8.2780
4F2B	0.0007	0.0410	0.0101	5.6073
4F2C	0.0034	0.0070	0.0055	1.8596
4F3A	0.0006	0.0397	0.0082	4.5000
4F3B	0.0011	0.0717	0.0122	0.0001

**Table 3:** The CNN network architecture used for the model development.

Layer Name	Output Size	Configuration
conv	64×112×112	7×7, 64, stride 2
max pool	64×56×56	3×3, maxpool, stride 2
conv block 1	256×56×56	$\left[\begin{array}{c} 1\times 1,\;64\\ 3\times 3,\;64\\ 1\times 1,\;256 \end{array}\right]\times 3$
conv block 2	512×28×28	$\left[\begin{array}{c} 1\times 1,\;64\\ 3\times 3,\;64\\ 1\times 1,\;256 \end{array}\right]\times 4$
conv block 3	1024×14×14	$\left[\begin{array}{c} 1\times 1,\;64\\ 3\times 3,\;64\\ 1\times 1,\;256 \end{array}\right]\times 6$
conv block 4	2048×7×7	$\left[\begin{array}{c} 1\times 1,\;64\\ 3\times 3,\;64\\ 1\times 1,\;256 \end{array}\right]\times 3$
global average pool	2048×1×1	-
fully connected	21	2048×21

**Table 4:** Performance of the trained models with different combinations of data augmentation techniques. (A) The performance of the model trained with all the data augmentation techniques except the addition of normal images. (B) The performance when only copy-pasting was not used. (C) The performance when only scale jittering was not used. (D) The performance when only translation was not used. (E) The performance when only cut-out was not used. (F) the performance when all the augmentation techniques were used.

	(A)	(B)	(C)	(D)	(E)	(F)
Normal images		✓	✓	✓	✓	✓
Copy-pasting	✓		✓	✓	✓	✓
Scale jittering	✓	✓		✓	✓	✓
Translation	✓	✓	✓		✓	✓
Cut-out	✓	✓	✓	✓		✓
mAP	0.9450	0.9477	0.9479	0.9599	0.9667	0.9571

**Table 5:** Type-wise performance of the developed deep learning model. $F1_{\theta =0.5}$ is the F1-score evaluated at a threshold 0.5 and AP is the average precision.

Fracture Type	$F1_{\theta =0.5}$	AP	Fracture Type	$F1_{\theta =0.5}$	AP
41A1	0.8571	1.0000	43A1	0.9388	0.9912
41A2	0.8750	0.9952	43A2	0.9538	0.9922
41A3	0.9655	1.0000	43A3	0.8282	0.9440
42A1	0.8901	0.9671	4F1A	0.7931	0.8852
42A2	0.9254	0.9831	4F1B	0.8824	0.9723
42A3	0.9630	1.0000	4F2A	0.7789	0.9124
42B2	0.8276	0.8874	4F2B	0.9193	0.9658
42B3	0.7857	0.9300	4F2C	1.0000	1.0000
42C2	0.8095	0.9497	4F3A	0.8852	0.9371
42C3	0.8462	0.9546	4F3B	0.8679	0.8748
average $F1_{\theta =0.5}$		0.8796			
mAP		0.9571			

**Table 6:** Performance of ResNet-50 and other advanced models.

Model	# Params	mAP
ResNet-50	23,551,061	**0.9571**
SE-ResNet-50	26,066,005	0.9430
CBAM-ResNet-50	26,083,653	0.9381
EfficientNet-B4	17,586,269	0.9514
EfficientNet-B5	28,383,813	0.9419
EfficientNet-B7	63,840,741	0.9018
ViT-S/8	21,678,357	0.7629
ViT-B/16	85,814,805	0.7354
Swin-T	27,535,503	0.9236
Swin-B	86,764,749	0.9038

## Data Availability

The data set is under IRB permission. Hence the data set cannot be disclosed in public.

## Abbreviations

The following abbreviations are used in this manuscript:

2D	Two-Dimensional
3D	Three-Dimensional
AI	Artificial Intelligence
AO/OTA	Arbeitsgemeinschaft für Osteosynthesefragen/Orthopedic Trauma Association
CNN	Convolutional Neural Network
CT	Computed Tomography
mAP	Mean Average Precision
MRI	Magnetic Resonance Imaging

## Appendix A

**Table A1:** The AO/OTA codes with their description.

AO/OTA Code	Code Description
41A1	a simple spiral [1] extra-articular [A] fracture of the proximal tibia [41]
41A2	a wedge-shaped [2], extra-articular [A] fracture of the proximal tibia [41]
41A3	a complex [3] extra-articular [A] fracture of the proximal tibia [41]
42A1	a simple [A] spiral [1] fracture of the tibial shaft [42]
42A2	a simple [A] oblique [2] fracture of the tibial shaft [42]
42A3	a simple [A] transverse [3] fracture of the tibial shaft [42]
42B2	an oblique [2] wedge [B] fracture of the tibial shaft. [42]
42B3	a transverse [3] wedge [B] fracture of the tibial shaft [42]
42C2	a complex [C], oblique fracture of the tibial shaft [42] with comminution[2]
42C3	a complex [3], multi-fragmentary segmental [3] fracture of the tibial shaft [42]
43A1	a simple spiral [1] extra-articular [A] fracture of the distal tibia [43]
43A2	an extra-articular [A], simple oblique [A] fracture of the distal tibia [43]
43A3	an extra-articular [A], simple transverse [3] fracture of the distal tibia [43]
4F1A	a simple [1], extra-articular [A] fracture [F] of the patella (bone of the knee) [4]
4F1B	a simple [1], intra-articular [B] fracture [F] of the patella [4]
4F2A	a multi-fragmentary [2], extra-articular [A] fracture [F] of the patella [4]
4F2B	a multi-fragmentary [2], intra-articular [B] fracture [F] of the patella [4]
4F2C	a multi-fragmentary [2], complex intra-articular [C] fracture [F] of the patella [4]
4F3A	a severely displaced [3], extra-articular [A] fracture [F] of the patella [4]
4F3B	a severely displaced [3], intra-articular [B] fracture [F] of the patella [4]
